# Sparking Fire Under the Skin? Answers From the Association of Complement Genes With Pemphigus Foliaceus

**DOI:** 10.3389/fimmu.2018.00695

**Published:** 2018-04-09

**Authors:** Valéria Bumiller-Bini, Gabriel Adelman Cipolla, Rodrigo Coutinho de Almeida, Maria Luiza Petzl-Erler, Danillo Gardenal Augusto, Angelica Beate Winter Boldt

**Affiliations:** ^1^Laboratory of Human Molecular Genetics, Department of Genetics, Universidade Federal do Paraná, Curitiba, Brazil; ^2^Departamento de Ciências Biológicas, Universidade Estadual de Santa Cruz, Ilhéus, Brazil; ^3^Laboratory of Molecular Immunopathology, Department of Clinical Pathology, Hospital de Clínicas, Universidade Federal do Paraná, Curitiba, Brazil

**Keywords:** pemphigus foliaceus, complement, lectin pathway, acantholysis, membrane attack complex, alternative pathway, opsonin, complement receptors

## Abstract

Skin blisters of pemphigus foliaceus (PF) present concomitant deposition of autoantibodies and components of the complement system (CS), whose gene polymorphisms are associated with susceptibility to different autoimmune diseases. To investigate these in PF, we evaluated 992 single-nucleotide polymorphisms (SNPs) of 44 CS genes, genotyped through microarray hybridization in 229 PF patients and 194 controls. After excluding SNPs with minor allele frequency <1%, out of Hardy–Weinberg equilibrium in controls or in strong linkage disequilibrium (*r*^2^ ≥ 0.8), 201 SNPs remained for logistic regression. Polymorphisms of 11 genes were associated with PF. *MASP1* encodes a crucial serine protease of the lectin pathway (rs13094773: OR = 0.5, *p* = 0.0316; rs850309: OR = 0.23, *p* = 0.03; rs3864098: OR = 1.53, *p* = 0.0383; rs698104: OR = 1.52, *p* = 0.0424; rs72549154: OR = 0.55, *p* = 0.0453). *C9* (rs187875: OR = 1.46, *p* = 0.0189; rs700218: OR = 0.12, *p* = 0.0471) and *C8A* (rs11206934: OR = 4.02, *p* = 0.0323) encode proteins of the membrane attack complex (MAC) and *C5AR1* (rs10404456: OR = 1.43, *p* = 0.0155), a potent anaphylatoxin-receptor. Two encode complement regulators: MAC-blocking CD59 (rs1047581: OR = 0.62, *p* = 0.0152) and alternative pathway-blocking CFH (rs34388368: OR = 2.57, *p* = 0.0195). One encodes opsonin: *C3* (rs4807895: OR = 2.52, *p* = 0.0239), whereas four encode receptors for C3 fragments: *CR1* (haplotype with rs6656401: OR = 1.37, *p* = 0.0382), *CR2* (rs2182911: OR = 0.23, *p* = 0.0263), *ITGAM* (CR3, rs12928810: OR = 0.66, *p* = 0.0435), and *ITGAX* (CR4, rs11574637: OR = 0.63, *p* = 0.0056). Associations reinforced former findings, regarding differential gene expression, serum levels, C3, and MAC deposition on lesions. Deregulation of previously barely noticed processes, e.g., the lectin and alternative pathways and opsonization-mediated phagocytosis, also modulate PF susceptibility. The results open new crucial avenues for understanding disease etiology and may improve PF treatment through additional therapeutic targets.

## Introduction

Pemphigus are blistering autoimmune diseases causing painful bullous lesions, resulting from keratinocyte detachment (acantholysis), through the loss of desmosomes (1). In pemphigus foliaceus (PF), they occur in the superficial granular layer, affecting the skin. Yet in pemphigus vulgaris (PV), they locate in suprabasal stratum, also damaging mucosa. Lesions’ localization correlate with tissue distribution of the main antigens: desmoglein 1 (DSG1) in PF and DSG3 in PV (2). Non-lesional skin may present blisters in the subgranular spinous layer, when submitted to mechanical friction (Nikolsky’s sign) (3). Epithelial PF lesions may be restricted to sun-exposed seborrheic trunk and head areas (localized form) or be ubiquitously distributed (generalized form) (4, 5).

### PF—An Epidemiological and Etiopathological Puzzle

Pemphigus occurs sporadically around the world, with incidence of 0.75–5 cases/million per year (6, 7). Despite this, PF is the only autoimmune disease known to be endemic in certain regions, as South America and Tunisia (5, 8), but epidemiology is puzzling, exhibiting wide differences even in neighboring countries. Midwestern Brazilian Amerindian populations actually present prevalences as high as 3.04% (9, 10). There is no sexual disproportion for PF in Brazil; most patients are young (10–40 years old) and have affected relatives. In Colombia, PF affects male mine workers and post-menopausal women (11), whereas young women are predominantly affected in Tunisia (9 female:1 male) (8). Endemic and non-endemic PF are indistinguishable (12), with the exception of higher anti-DSG1 IgM and IgE serum levels in endemic PF (13–15).

The epidemiological puzzle adds to the lack of understanding regarding PF etiology, since postulated major causes differ among countries (11). Brazilian PF patients are usually rural low-wage workers (4), exposed to acantholysis-fostering factors such as UVB (16), thiol and other calcium-sequestering components (11). Most present frequent bites of black (Simuliidae) and sand flies (Phlebotominae), vectors of onchocerciasis and leishmaniasis, respectively. Bites were suggested to increase up to almost five times the susceptibility to PF (17, 18), and components of the fly saliva may trigger a cross-reaction against keratinocyte surface epitopes (19). Viral or bacterial etiology was also suggested (5, 11). Genetic susceptibility involves differential gene expression (20, 21), variants in genes encoding antigen-presenting molecules HLA-DR and HLA-DQ (22, 23) and their corresponding regulatory transcription factor CIITA (24). Several other associations with genes of the immune response have been reported (25–31).

Pemphigus foliaceus patients have higher serum immunoglobulin G (IgG) levels against desmocollins 1 and 2 and all four desmogleins (32). Most pathogenic antibodies, able to induce acantholysis *in vitro* and *in vivo* (33–35), are of the IgG4 subclass (36–38), directed against the DSG1 N-terminal ectodomains (39, 40). Anti-DSG1 IgG1 are common in asymptomatic individuals of endemic regions, but can be the only pathogenic antibodies in a subset of PF patients (19). In contrast to IgG4, they initiate the classical pathway of the complement system (CS). This agrees with the frequent concomitant deposition of antibodies and CS components in PF lesions (41–45). Administration of corticosteroids is crucial to achieve disease control in the acute stage. Due to numerous and severe side effects, pemphigus patients are in desperate need of new, specifically targeted therapeutic strategies to substitute common therapy [reviewed in Ref. (46)].

### PF and Complement: A Controversial Issue

Complement includes more than 50 plasma and membrane-bound proteins working in the forefront of host defense, killing pathogens and altered cells, and connecting innate to adaptive immune responses. Classical activation begins with the recognition of IgG or IgM, molecules on microbial and apoptotic cells, and C-reactive protein (CRP) by the C1 complex (C1q complexed with serine proteases C1r and C1s). The alternative activation pathway unleashes by spontaneous proteolysis of component C3. The lectin pathway follows recognition of sugar moieties or acetylated residues by colectins (as mannose-binding lectin—MBL) or ficolins (FCNs), respectively, complexed with another set of serine proteases (MASP-1 and MASP-2) (47, 48). All pathways converge in the formation of C3 convertase, which produces opsonic fragments that enhance antigen clearance by phagocytosis. C3b opsonin may be incorporated in the C5 convertase, which leads to the release of C5a anaphylatoxins and to pores opening on target cells, by insertion of the membrane attack complex (C5b-9 complex or MAC). Recognition of CS fragments leads to phagocytosis or blockage of the cascade, which is constantly activated at low levels, being continuously controlled to avoid tissue damage. Far beyond these well-known roles, CS also accomplishes critical functions in regulating inflammation, nervous system development and maturation, coagulation and hemostasis (49, 50).

In a series of five 1980s articles, entitled “Complement fixation by pemphigus antibody,” the Jordon’s group chased the hypothesis that complement has an important role in PV blister formation (51–55). They were closely followed by others who argued the same for PF. Strong granular C3 deposition was repeatedly reported along the basement membrane zone and in intercellular spaces of the epidermal strata (41–45). C3 was also reported to colocalize with IgG1 deposits in the upper epidermis intercellular spaces, in intact as well as injured skin, with a trend for higher deposits in perilesional tissue (42, 43, 45, 56). In fact, C1q and C4 fragments (reported in one patient), and MAC deposits distinguish injured skin, since IgG4 also occurs abundantly in non-acantholytic tissue (42, 43). In cell culture, complement does not seem necessary for acantholysis, but enhances keratinocyte detachment (55, 57).

Serum levels of C3 and CRP (opsonins), Ba and C4d factors (indicative of activated alternative and classical/lectin pathways, respectively), are increased in PF patients with active disease (58–60). CD4^+^ T cells of PF patients present upregulated *C1QA* gene expression, compared to controls (20). Protein levels and *C1QA* expression fall with therapeutic intervention (20, 59, 60). By contrast, anti-DSG1 IgG levels remain high during disease remission (61, 62). MASP-2 levels tend to decline in PF patients, but MBL serum concentrations seem unaffected (63). In PV biopsies, MBL and FCN2, but not C1q nor FCN3, recognize antigens in the basal membrane zone and intercellular spaces of the epidermis (64).

Nevertheless, C5-deficient mice or complement-depleted mice (after inoculation with cobra venom factor) develop the disease when injected with non-endemic PF IgG4 or its F(ab′)2 fragments (65). Both models did not affect C3 upstream components of the classical and lectin pathways, meaning that any roles played by these initiator molecules in the acantholytic process were not appreciated. In addition, the abundant but non-pathogenic anti-DSG1 human IgG1 does not cross-react with murine epidermis (66). By contrast, anti-DSG3 autoantibodies of PF patients, with cutaneous disease only, induce PV-like lesions in mice (67). Conversely, anti-DSG1 autoantibodies of PV patients without superficial epithelial lesions induce PF-like lesions (68). Adding to this picture, *DSG* expression pattern greatly differs between human and mouse (69) and differences in the genetic background of mouse models, which may deliver completely different outcomes for cutaneous inflammation, were not accounted for (70). Thus, although the mouse model reproduces acantholysis, it cannot reproduce the natural history of the disease itself, and pathological mechanisms may be quite different. For example, murine lesions present apoptotic cells (71), an uncommon finding in human biopsies (3, 72–74), with one reported exception (75). These results undeniably places complement in the disease, but its possible roles are still an issue to be solved.

## Genetic Association Between Complement Genes and PF

In the late 90s, the observations from experimental models seemed to have settled the interest on the role of complement in pemphigus. Nevertheless, tissue damage and inflammation, through over-activation and/or deficiency of complement components, play a key role in many dermatological diseases (76). These host-offensive actions may be exacerbated by genetic variation (77), but the extensive polymorphism of complement components impairs the comprehension of their overall impact in any given disease. In addition to the great genetic variation, the pleiotropic effects observed for complement genes add another layer of complexity.

Knowing that genetic associations may reveal new elements that play pivotal roles in disease susceptibility, we intend to reignite this discussion with new results of a PF case–control study that encompass tag polymorphisms within CS genes (Table S1 in Supplementary Material). We analyzed 992 single-nucleotide polymorphisms (SNPs) distributed within 44 genes, out of a subset of 551,839 SNPs genotyped in 229 endemic PF patients and 194 controls, through microarray hybridization (CoreExome-24 v1.1 Illumina). Included patients presented confirmed clinical PF diagnosis, according to physical examination and immunohistochemistry results. Controls were individuals of the endemic region, with no diagnosis or familial history of autoimmune diseases and unrelated to the patients. This study was carried out in accordance with the recommendations of the guidelines of the Conselho Nacional de Ética em Pesquisa (CONEP) with written informed consent from all subjects. All subjects gave written informed consent in accordance with the Declaration of Helsinki. The protocol was approved by CONEP (number 505.988). The statistical analyses were done with PLINK v1.1.9 (78). After excluding those SNPs with minor allele frequencies >1%, genotypic distributions deviating from those expected by Hardy–Weinberg equilibrium in controls (*p* < 0.05) and high linkage disequilibrium (*r*^2^ ≥ 0.8), 201 SNPs remained for subsequent analyses. For haplotypic analysis, 35 additional SNPs with *r*^2^ > 0.8 were included. Association analysis was carried out by binary logistic regression, using two principal components (PCA) as covariables, which efficiently eliminates spurious associations due to ethnical differences. Thus, significance level was set to *p* = 0.05. Rather than exhausting the debate, our purpose is to launch new hypotheses that could be further validated through functional studies, which will link the pathogenic role of the PF autoantibodies to CS underexplored arms.

## The Long Known Versus the Unexpected: Complement in PF

**Figure 1:**
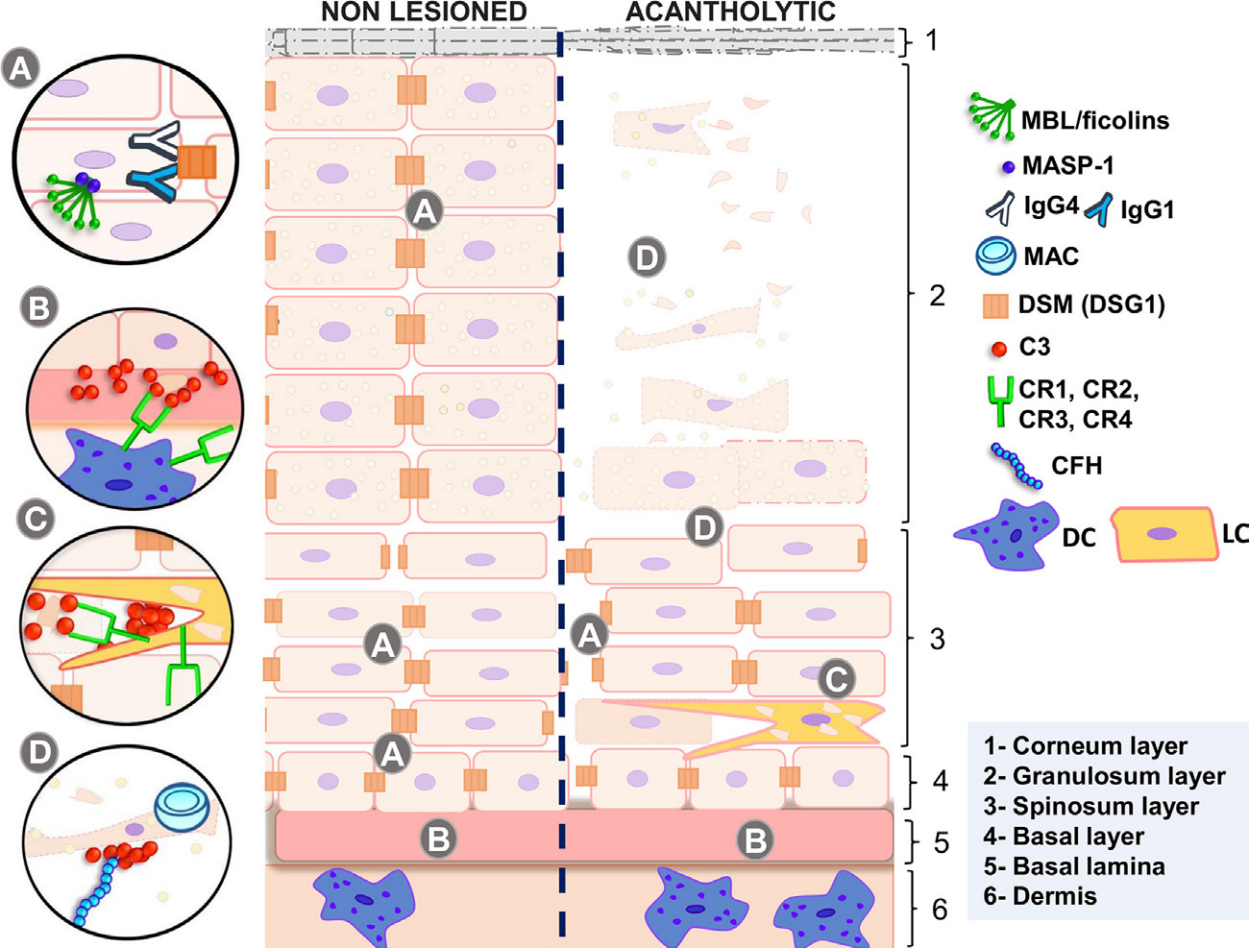
Complement in non-lesioned and acantholytic PF lesions. **(A)** IgG1 and IgG4 autoantibodies binding to desmosomes causes shrinkage of keratinocytes, increasing intercellular spaces. This process is fostered by activation of the p38 MAPK signaling cascade, which may be unleashed by MASP-1, the serine protease associated with initiating molecules of the lectin pathway. **(B)** This is further accompanied by the release of antigens, most probably recognized by MBL or ficolins, leading to granular deposition of C3 fragments in the basal lamina. These deposits, as well as deposits of C3 fragments in the intercellular spaces, may also be caused by activation of the alternative pathway, inhibited by CFH. **(C)** Complement receptors recognize C3 fragments, leading to phagocytosis of autoantigens and increasing antigen presentation to T lymphocytes, thus feedbacking and diversifying autoantibody production. **(D)** Acantholytic lesions present formation of the membrane attack complex (blocked by CD59 expression), which may protect cells against apoptosis, if present in sublytic amounts. Active disease is also followed by increased C5a release, the latter recognized by C5A receptors in dendritic cells. MBL, mannose-binding lectin; MASP-1, mannose-binding lectin serine protease 1; MASP-2, mannose-binding lectin serine protease 2; MAC, membrane attack complex; IgG4, immunoglobulin 4; IgG1, immunoglobulin 1; DSM (DSG1), desmosome (desmoglein 1); C3, complement component 3; CR1, complement receptor type 1; CR2, complement receptor type 2; CR3, complement receptor type 3; CR4, complement receptor type 4; CFH, complement factor H; DC, dendritic cell; LC, Langerhans cell; PF, pemphigus foliaceus. Source: the author (2018).

We found evidence of association with gene variants of almost all complement elements previously detected in the epidermis or with altered serum levels in PF patients (Table 1; Figure 1). Among them, homozygotes for the intronic rs4807895*T allele within the *C3* gene were more susceptible to the disease (OR = 2.52; *p* = 0.0239). C3 fragments have been consistently reported in PF lesions (41–45, 56, 79, 80) and necessarily result from the activation of proteolytic cascades that converge in its enzymatic cleavage. Given the lack of functional evidence for this association, we speculate that it could be partly explained by increased *C3* gene regulation. This would not only increase phagocytosis and MAC deposition, but also T cell-mediated skin inflammation, as reported in other autoimmune diseases (81, 82).

**Table 1:** Complement gene variants associated with PF.

Gene	SNP	eQTL	Direction		MAF (%)		Model	Contr	Pat	OR	95 % CI	*p*
				Ib	Contr	Pat						
*C3*	rs4807895	–	–	30.8	25.26	29.04	add	98/290	133/325	1.26	[0.93–1.72]	0.1377
19p13.3	*t>C*						**rec**	**9/185**	**23/206**	**2.52**	**[1.13**–**5.62]**	**0.0239**
	Intron 11						dom	89/105	110/119	1.14	[0.77–1.67]	0.5177
*C5AR1*	rs10404456	3.2 × 10–^5^*a	Down	25.2	31.87	40.67	**add**	**123/263**	**183/267**	**1.43**	**[1.07**–**1.90]**	**0.0155**
19q13.32	*c*>*T*						rec	20/173	38/187	1.67	[0.93–3.00]	0.0836
	5′ UTR						**dom**	**103/90**	**145/80**	**1.55**	**[1.04**–**2.30]**	**0.0299**
*C8A*	rs11206934	–	–	22	17.62	19.38	add	68/318	88/366	1.15	[0.81–1.63]	0.4228
1p32.2	*T>c*						**rec**	**3/190**	**13/214**	**4.02**	**[1.12–14.3]**	**0.0323**
	Intron 10						dom	65/128	75/152	1.00	[0.66–1.51]	0.993
*C9*	rs700218	–	–	0.9	2.10	0.21	**add**	**8/380**	**1/457**	**0.12**	**[0.01**–**0.97]**	**0.0471**
5p13.1	*G>t*						rec	0/194	0/229	–	–	–
	Intron 1						**dom**	**8/186**	**1/228**	**0.12**	**[0.01**–**0.97]**	**0.0471**
	rs187875	6.5 × 10^−6b^	Up	31.8	23.26	30	**add**	**87/287**	**132/308**	**1.46**	**[1.06–2.01]**	**0.0189**
	*C>t*						rec	10/177	23/197	2.13	[0.98–4.64]	0.0556
	Intron 6						dom	77/110	109/111	1.49	[1.00–2.23]	0.0509
*CD59*	rs1047581	7.3 × 10^−9a^	Down	40.2	31.19	23.8	**add**	**121/267**	**109/349**	**0.72**	**[0.52**–**0.98]**	**0.0373**
11p13	*A>g*						rec	16/178	15/214	0.87	[0.41–1.83]	0.7166
	3′ UTR						**dom**	**105/89**	**94/135**	**0.62**	**[0.42**–**0.91]**	**0.0152**
*CFH*	rs34388368	7.1 × 10^−7b^	Up	22.4	27.13	29.68	add	102/274	130/308	1.11	[0.81–1.50]	0.5185
1q31.3	*G>t*						**rec**	**9/179**	**25/194**	**2.57**	**[1.16**–**5.66]**	**0.0195**
	Intron 1						add	93/95	105/114	0.90	[0.61–1.34]	0.6075
*CR2*	rs2182911	–	–	18.2	19.85	18.72	add	77/311	85/369	0.93	[0.65–1.32]	0.6813
1q32.2	*c>T*						**rec**	**11/183**	**3/224**	**0.23**	**[0.06**–**0.84]**	**0.0263**
	Intron 19						dom	66/128	82/145	1.09	[0.73–1.64]	0.6644
*ITGAM*	rs12928810	–	–	25.9	23.7	19.56	add	91/293	88/362	0.74	[0.53–1.03]	0.0735
16p11.2	*G>a*						rec	76/150	12/213	0.84	[0.36–1.98]	0.693
	Intron 14						**dom**	**80/112**	**76/149**	**0.66**	**[0.44**–**0.99]**	**0.0435**
*ITGAX*	rs11574637	–	–	22.9	28.09	20	**add**	**109/279**	**90/360**	**0.63**	**[0.45**–**0.87]**	**0.0056**
16p11.2	*T*>c						rec	14/180	10/215	0.55	[0.23–-1.29]	0.1698
	Exon 4						**dom**	**95/99**	**80/145**	**0.57**	**[0.39**–**0.85]**	**0.0058**
*MASP1*	rs13094773	4.6 × 10^−8a^	Down	35	34.9	30.22	add	134/250	136/314	0.82	[0.61–1.09]	0.1747
3q27.3	*A>g*						**rec**	**28/164**	**18/207**	**0.50**	**[0.27–0.94]**	**0.0316**
	Intron 1						dom	106/86	118/107	0.91	[0.62–1.35]	0.6479
	rs3864098	3.5 × 10^−8a^	Up	15	17.53	22.22	add	68/320	102/356	1.34	[0.94–1.92]	0.1066
	*T*>c						rec	7/187	6/223	0.68	[0.22–2.09]	0.5043
	Intron 2						**dom**	**61/133**	**96/133**	**1.53**	**[1.02**–**2.29]**	**0.0383**
	rs698104	2.2 × 10^−10a^	Up	14	20.62	26.86	add	80/308	123/335	1.36	[0.96–1.90]	0.0787
	*t>C*						rec	9/185	13/216	1.08	[0.44–2.62]	0.8698
	Intron 2						**dom**	**71/123**	**110/119**	**1.52**	**[1.01**–**2.26]**	**0.0424**
	rs850309	1.1 × 10^−18b^	Up	19.2	20.68	21.78	add	79/303	98/352	1.06	[0.74–1.50]	0.7596
	*A>g*						**rec**	**10/181**	**3/222**	**0.23**	**[0.06**–**0.87]**	**0.03**
	Intron 3						dom	69/122	95/130	1.27	[0.85–1.90]	0.2357
	rs72549154	–	–	2.8	7.51	4.64	**add**	**29/357**	**21/431**	**0.55**	**[0.31**–**0.99]**	**0.0453**
	*G>t*						rec	3/190	0/226	–	–	–
	Exon 12						dom	26/167	21/205	0.60	[0.32–1.12]	0.1119

*Logistic regression association tests were done with allele frequencies (“add”), frequency of homozygotes for the minor allele (“rec”—recessive model), and summed frequency of heterozygotes and homozygotes for the minor allele (“dom”—dominant model). The minor allele is given in lowercase*.

*In bold: significant results; SNP, single-nucleotide polymorphism; eQTL, expression quantitative trait loci (83)*.

*^a^In skin*.

*^b^In hypodermis, direction: increase or decrease of RNA expression in relation the MAF*.

*MAF, minor allele frequency; Ib, Iberian population (84); Contr, controls; Pat, patients, Model, association tests; OR, odds ratio; CI, confidence interval; PF, pemphigus foliaceus*.

*ENSEMBL 2018 http://www.ensembl.org/index.html, GTEx portal—https://www.gtexportal.org/home/*.

Among the pathways held responsible for generating C3 fragments, we found association with genetic variants within genes of the alternative and lectin pathways, but not with the classical pathway. This agrees with the almost complete absence of C1q in human biopsies (42, 64). It also argues against the traditional hypothesis that activation of the classical pathway by anti-DSG1 IgG1 would play an important role in PF (42, 52, 85). In fact, the most abundant pathogenic IgG subclass in pemphigus is IgG4, which is unable to activate complement (33, 34).

Regarding the alternative pathway, we found a surprising genetic association with factor H, its most important regulator. Homozygotes for CFH rs34388368*T, an intronic allele associated with higher *CFH* mRNA levels in the hypodermis (83), were more susceptible to PF (OR = 2.57; *p* = 0.0195). These results contradict the conception that uncontrolled complement activation would be one of the underlying causes of PF.

We also found association between PF and five *MASP1* polymorphisms, four of them associated with differential mRNA levels in sun-exposed skin and/or in the hypodermis (83). They can potentially interfere with alternative pre-mRNA splicing, which generates three *MASP1* products—the collectin/ficolin-associated serine proteases 1 and 3 (MASP-1 and MASP-3) and the truncated non-catalytic MAp44 (also called MAp1, only expressed in cardiac tissue). These products play important roles in competitive activation and blockage of the lectin and alternative pathways, intracellular signaling, coagulation, and bradykinin/kinin systems (86). In our setting, homozygotes for rs13094773*G and rs850309*G (within an intronic region recognized by multiple regulatory proteins) (87) were more protected against PF (OR = 0.5; *p* = 0.0316 and OR = 0.23; *p* = 0.03, respectively). Yet individuals with intronic rs3864098*C (OR = 1.53; *p* = 0.0383) or rs698104*T (OR = 1.52; *p* = 0.0424) presented increased susceptibility to the disease. Of note, the rs3864098*C allele occurs in linkage disequilibrium with rs710469*C, an allele associated with lower MASP-3 levels in pre-admission critically ill children (88) (Table S2 in Supplementary Material). Finally, we found a protective association (OR = 0.55; *p* = 0.0453) with a missense variant affecting exclusively the serine protease domain of MASP-3 (rs72549154*T in exon 12, encoding p.Arg576Met). Heterozygotes for rs72549154*T present proportionally increased MASP-3 and decreased MASP-1 serum levels (89). Co-occurring *MASP1* alleles increase susceptibility to PF (not necessarily within the same haplotype): rs13094773*A combined with rs3864098*C (OR = 2.51 [95% CI = 1.26–4.97], *p* = 0.0063), rs13094773*A, and rs698104*T (OR = 2.37 [95% CI = 1.22–4.59], *p* = 0.0074) and between rs3864098*C and rs698104*T (OR = 1.67 [95% CI = 1.09–2.55], *p* = 0.0141). All the three variants are associated with higher MASP1 levels (83). Thus, it is conceivable that higher MASP-1 levels contribute to PF, while higher MASP-3 levels are protective. From the physiological point of view, altered MASP-1 levels would affect activation of the lectin pathway, which relies entirely on MASP-1 autoactivation (90). Additionally, MASP-1 activates MASP-3, which cleaves pro-factor D and launches the alternative complement cascade under non-inflammatory conditions (91). It further activates the p38 MAPK pathway in endothelial cells, which leads to IL-8 secretion and neutrophil recruitment (92, 93), both reported to occur in different forms of pemphigus (94–96). Most importantly, activation of the p38 MAPK signaling cascade causes acantholysis in keratinocytes and may be initiated by MASP-1 as well (97, 98).

Genetic variants of MAC components were also associated with PF. Homozygotes for the less common *C8A* allele rs11206934*C (OR = 4.02; *p* = 0.0323) in intron 10 and individuals with a *C9* haplotype harboring intronic variants consistently associated with increased gene expression in hypodermis and mucosa—rs187875*T (which disrupts a methylated CpG) (87) presented higher susceptibility to PF (Table S2 in Supplementary Material). By contrast, individuals with rs700218*A (intron 1 of *C9*) were more protected (OR = 0.12; *p* = 0.0471). We found no association with *C5* polymorphisms, as reported by others (who investigated only one SNP) (99). Nevertheless, individuals with the rs10404456*C allele (located in the 5′ UTR of the *C5AR1* gene and associated with decreased mRNA levels in sun-exposed skin) (83) presented increased susceptibility to PF (OR = 1.43 *p* = 0.0155). This gene encodes the major receptor for C5a anaphylatoxin (49) and its deficiency has been rather associated with protection against several immune complex-mediated diseases, including epidermolysis bullosa acquisita (70, 100).

Keratinocytes may keep MAC formation at sublytic levels, eliciting pro-survival signal transduction, hence inhibiting apoptosis—instead of promoting cell destruction (101). This may be achieved by expressing low *CD59* levels, MAC’s most important inhibitor. In fact, rs1047581*G in the 3′UTR region of the *CD59* gene, associated with reduced mRNA levels in sun-exposed skin (83), protected against PF (OR = 0.62; *p* = 0.0152). This result agrees with a recent study of our group, where the alternative allele of this same polymorphism occurs within a haplotype increasing *CD59* mRNA expression and PF susceptibility (32).

Among complement main roles, the removal of immune complexes and cellular debris is of critical importance for autoimmunity prevention (70). Within the context of the other associations, we suggest that protection may be explained by higher scavenging efficiency of acantholytic cell debris. Furthermore, we found associations with four opsonin-binding complement receptors (CR1-4, encoded by *CR1, CR2, ITGAM*, and *ITGAX*). Interestingly, we found a susceptibility association with a *CR1* haplotype that includes the major rs6656401*G allele (Table S2 in Supplementary Material), also associated with protection against Alzheimer’s disease (102). The binding of CR2 to iC3b, C3dg, and C3d lowers the threshold for B cell activation (103) and homozygotes for rs2182911*C of the *CR2* gene were more protected against the disease (OR = 0.23; *p* = 0.0263). The products of *ITGAM* (CR3) and *ITGAX* (CR4) genes recognize iC3b (48). Individuals with the rs12928810*A (disrupts a CpG in intron 14 of *ITGAM*) or rs11574637*C (a missense variant—p.Phe180Leu—in exon 4 of *ITGAX*) were more resistant against PF (OR = 0.66; *p* = 0.0435 and OR = 0.63; *p* = 0.0056, respectively). Remarkably, the same *ITGAX* allele was associated with higher susceptibility to IgA nephropathy and systemic lupus erythematosus (104, 105). The rs11574637*C (*ITGAX*) and rs4807895*T (*C3*) combined are protective against PF (OR = 0.55 [95% CI = 0.32–0.95], *p* = 0.0276). The same occurs with the rs11574637*C (*ITGAX*) and the rs12928810*A (*ITGAM*) (OR = 0.59 [95% CI = 0.38–0.90], *p* = 0.0115). By contrast, individuals presenting both the rs10404456*C (*C5AR1*) and rs12928810*G/G (*ITGAM*) are more susceptible to PF (OR = 2.33 [95% CI = 1.27–4.28], *p* = 0.0035), as were those with rs10404456*C (*C5AR1*) and rs11574637*T/T (*ITGAX*) (OR = 2.64 [95% CI = 1.49–4.66], *p* = 0.0006). In a previous study of our group, the mRNA expression levels of *ITGAM* were increased in CD4^+^ T cells of PF patients with generalized lesions, whereas *ITGAX* mRNA expression decreased after treatment (20).

## Perspectives

Complement gene associations reinforced the findings of former studies, regarding the alternative pathway, C3 and MAC deposition on epidermal cells. Our results shed light on previously barely noticed processes, notably CS-mediated signaling, especially by MASP-1, and removal of opsonized elements, through complement receptors. The role of antigen-presenting phagocytes bearing CR1, CR2, CR3, and CR4, as dendritic and Langerhans cells, should be deeper investigated, since they probably exert crucial roles in the events preceding B cell activation and autoantibody production. Furthermore, lectin and alternative pathways, activated at low levels, are probably important to prevent the disease. Taken together, the results on these pathways lead us to suggest caution on the possible use of the two available complement-inhibiting drugs, able to prevent classical/lectin pathway initiation (C1INH) and MAC generation (Eculizumab), since complement activation appears desirable to PF prevention. Strong evidence for *MASP1* association, but not for *MASP2* or other genes of the lectin pathway, favor a pathogenic role carried out by MASP-1 in eliciting p38MAPK signaling and consequent Dsg1 clustering on the keratinocyte cell membrane. Functional validation of the pathogenic roles exerted by this wide-reaching network of complement components will open new windows to understand PF etiology and development, hopefully improving therapeutic interventions.

## Ethics Statement

This study was carried out in accordance with the recommendations of the guidelines of the Conselho Nacional de Ética em Pesquisa (CONEP) with written informed consent from all subjects. All subjects gave written informed consent in accordance with the Declaration of Helsinki. The protocol was approved by CONEP (number 505.988).

## Author Contributions

MP-E, AB, GC, RA, and DA contributed to conception of the work. AB and VB-B designed the study. MP-E provided the samples. DA performed microarray hybridization. VB-B and RA did the statistical analysis. VB-B, AB, and GC drafted the manuscript. All authors revised the work critically for intellectual content and approved the final version of the work.

## Conflict of Interest Statement

The authors declare that the research was conducted in the absence of any commercial or financial relationships that could be construed as a potential conflict of interest. The reviewer HB and handling Editor declared their shared affiliation.
