# Supplementary material for: Sparking Fire Under the Skin? Answers From the Association of Complement Genes With Pemphigus Foliaceus
Source: Front Immunol. 2018 Apr 9;9:695. doi: 10.3389/fimmu.2018.00695 (PMC5900433; doi:10.3389/fimmu.2018.00695)
Supplement: Supplementary file 1 [file table_1.PDF]

Supplementary Material

Sparking fire under the skin? Answers from the association of complement genes with pemphigus foliaceus

Valéria Bumiller Bini, Rodrigo Coutinho de Almeida, Gabriel A. Cipolla, Maria Luiza Petzl-Erler, Danillo Gardenal Augusto, Angelica Beate Winter Boldt*

*** Correspondence:** Corresponding Author: angelicaboldt@gmail.com

Supplementary Table 1. Genes selected for evaluating SNP association with pemphigus foliaceus**.**

**Protein**	**Gene**	**Chr**	**Start**	**End**
Pattern recognition				
C1q	*C1QA*	1p36.12	22,962,118	22,967,175
Collectin11	*COLEC11*	2p25.3	3,641,422	3,693,234
MBL	*MBL2*	10q21.1	54,524,140	54,532,460
Ficolin-1	*FCN1*	9q34.3	137,800,431	137,810,806
Ficolin-2	*FCN2*	9q34.3	137,771,658	137,780,366
Ficolin-3	*FCN3*	1p36.11	27,694,601	27,702,315
Properdin	*CFP*	Xp11.23	47,482,612	47,490,704
CRPa	*CRP*	1q23.2	159,482,612	159,685,379
CFHR-4	*CFHR4*	1q31.3	196,658,182	196,888,843
Proteases				
C1r	*C1R*	12p13.31	7,186,515	7,246,043
C1s	*C1S*	12p13.31	7,166,980	7,179,335
MASP-1, MASP-3*	*MASP1*	3q27.3	186,950,870	187,010,810
MASP-2	*MASP2*	1p36.22	11,085,580	11,108,296
C2	*C2*	6p21.33	3,237,324	3,257,269
Factor B	*CFB*	6p21.33	3,227,026	3,253,571
Factor D	*CFD*	19p13.3	858,665	864,610
Factor I	*CFI*	4q25	110,660,848	110,724,335
Complement cascade components				
C3	*C3*	19p13.3	6,676,846	6,721,662
C4	*C4A*	6p21.33	31,980,167	31,984,434
C5	*C5*	9q33.2	123,713,614	123,813,554
C6	*C6*	5p13.1	41,141,927	41,204,332
C7	*C7*	5p13.1	40,908,599	40,984,042
C8	*C8A*	1p32.2	57,319,443	57,384,894
C8	*C8B*	1p32.2	57,393,883	57,432,688
C9	*C9*	5p13.1	39,284,301	39,365,566
Receptors				
CR1	*CR1*	1q32.2	207,668,473	207,816,110
CR2	*CR2*	1q32.2	207,626,645	207,664,240

CR3	*ITGAM*	16p11.2	31,270,288	31,345,213
CR4	*ITGAX*	16p11.2	31,365,509	31,395,264
C3aR	*C3AR1*	12p13.31	8,209,919	8,219,955
C5aR	*C5AR1*	19q13.32	47,812,153	47,825,087
C5L2	*C5AR2*	19q13.32	47,834,404	47,846,272
CRIg	*VSIG4*	Xq12	65,240,580	65,260,967
cC1qR	*CALR*	19p13.11	13,048,414	13,056,304
gC1qR	*C1QBP*	17p13.2	5,335,099	5,343,471
Regulators				
C1inh	*SERPING1*	11q12.1	57,364,027	57,383,326
MAP19 or sMAP	*MASP2*	1p36.22	11,085,580	11,108,296
MAp44 or MAP-1	*MASP1*	3q27.3	186,950,870	187,010,810
C4BP	*C4BPA*	1q32.2	207,276,607	207,319,317
Factor H	*CFH*	1q31.3	196,620,008	196,717,634
FHL-1	*CFHR1*	1q31.3	196,787,861	196,802,319
MCP	*CD46*	1q32.2	207,924,558	207,967,936
DAF	*CD55*	1q32.2	207,493,817	207,535,311
CFHR-1	*CFH*	1q31.3	196,620,008	196,717,634
CD59	*CD59*	11p13	33,723,556	33,745,273
Vitronectin	*VTN*	17q11.2	26,696,299	26,698,373
Clusterin	*CLU*	8p21.1	27,453,434	27,473,328
Carboxypeptidase-N	*CPN1*	10q24.2	101,801,065	101,842,642

We added 1000 bp to the start and end gene positions, to screen SNPs of regulatory sequences.

The full list of SNP results is available upon request from the corresponding author.

Reference sequence: GRCh37/hg19 * generated by alternative splicing Chr: chromosomal cytogenetic localization
