# Supplementary material for: Sparking Fire Under the Skin? Answers From the Association of Complement Genes With Pemphigus Foliaceus
Source: Front Immunol. 2018 Apr 9;9:695. doi: 10.3389/fimmu.2018.00695 (PMC5900433; doi:10.3389/fimmu.2018.00695)
Supplement: Supplementary file 2 [file table_2.PDF]

Supplementary Material

Sparking fire under the skin? Answers from the association of complement genes with pemphigus foliaceus

Valéria Bumiller Bini, Rodrigo Coutinho de Almeida, Gabriel A. Cipolla, Maria Luiza Petzl-Erler, Danillo Gardenal Augusto, Angelica Beate Winter Boldt*

*** Correspondence:** Corresponding Author: angelicaboldt@gmail.com

Supplementary Table 2. Complement gene haplotypes associated with Pemphigus foliaceus.

Genes	SNPs	Haplotype	Frequency (%)		OR	CI [95%]	*p*
			controls	patients			
CR1 (1q32.2)	rs11117956, rs6656401, rs12034598, rs3737002, rs3738468, rs17259045	*TGACGA*	27.12	33.17	1.60	[1.07-2.40]	0.021
MASP1 (3q27.3)	rs710469, rs3864098	*CC*	17.02	22.47	1.60	[1.05-2.42]	0.027
C9 (5p13.1)	rs155375, rs187875	*GT*	2.326	30.27	1.55	[1.04-2.32]	0.032
ITGAX (16p11.2)	rs11574637, rs2230429, rs11150619	*CGT*	28.09	20.0	0.57	[0.38-0.85]	0.006

Within parentheses: cytogenetic localization. SNP: single nucleotide polymorphism OR: Odds ratio, CI: confidence interval. OR, p-value and 95% CI was obtained with Stata v.9.2

CR1: Complement receptor 1; *MASP1*: Mannose-binding lectin serine protease 1; *C9*: Complement component 9; *ITGAX*: Integrin Subunit Alpha X (identical with complement receptor 4 or CR4).
